# Characterization and noninvasive diagnosis of bladder cancer with serum surface enhanced Raman spectroscopy and genetic algorithms

**DOI:** 10.1038/srep09582

**Published:** 2015-05-07

**Authors:** Shaoxin Li, Linfang Li, Qiuyao Zeng, Yanjiao Zhang, Zhouyi Guo, Zhiming Liu, Mei Jin, Chengkang Su, Lin Lin, Junfa Xu, Songhao Liu

**Affiliations:** 1 Biomedical Engineering Laboratory, School of Information Engineering, Guangdong Medical College, Dongguan 523808, Guangdong, China; 2 State Key Laboratory of Oncology in South China and Department of Clinical Laboratory, Sun Yat-sen University Cancer Center, Guangzhou, 510060, China; 3 School of Basic Medicine, Guangdong Medical College, Dongguan 523808, Guangdong, China; 4 MOE Key Laboratory of Laser Life Science & SATCM Third Grade Laboratory of Chinese Medicine and Photonics Technology, College of Biophotonics, South China Normal University, Guangzhou 510631, China; 5 Guangdong Provincial Key Laboratory of Medical Molecular Diagnostics, No. 1 Xincheng Road, Dongguan 523808, China

## Abstract

This study aims to characterize and classify serum surface-enhanced Raman spectroscopy (SERS) spectra between bladder cancer patients and normal volunteers by genetic algorithms (GAs) combined with linear discriminate analysis (LDA). Two group serum SERS spectra excited with nanoparticles are collected from healthy volunteers (n = 36) and bladder cancer patients (n = 55). Six diagnostic Raman bands in the regions of 481–486, 682–687, 1018–1034, 1313–1323, 1450–1459 and 1582–1587 cm^−1^ related to proteins, nucleic acids and lipids are picked out with the GAs and LDA. By the diagnostic models built with the identified six Raman bands, the improved diagnostic sensitivity of 90.9% and specificity of 100% were acquired for classifying bladder cancer patients from normal serum SERS spectra. The results are superior to the sensitivity of 74.6% and specificity of 97.2% obtained with principal component analysis by the same serum SERS spectra dataset. Receiver operating characteristic (ROC) curves further confirmed the efficiency of diagnostic algorithm based on GA-LDA technique. This exploratory work demonstrates that the serum SERS associated with GA-LDA technique has enormous potential to characterize and non-invasively detect bladder cancer through peripheral blood.

Urinary bladder cancer (BC) has become the fifth most common cancer worldwide. A recent study in the European Union reported the incidence of 19.5 cases and mortality of 7.9 cases per 100,000 populations. The incidence of BC varies globally with the lowest rates in Asia and the highest rates in Europe and America^1^,^2^. The recurrence rate of BC is very high and more than half recur within 5 years, therefore, early detection of BC is essential for preventing early recurrence and progression. Currently cystoscopy holds the gold standard to diagnose BC, but it is an invasive, unpleasant and expensive method. Voided urine cytology is a non-invasive means to identify BC, but it has limited sensitivity for low grade BC. Many urine-based biomarkers such as ImmunoCyt™, cytokeratin and telomerase have been developed, but they also show limited sensitivity for the detection of low grade BC^1^.

Recently Raman spectroscopy has attracted many attentions for its application in cancer detection^3^. Raman spectroscopy is a molecular vibration spectra technique discovered by Indian scientist Raman C.V. in 1928. It can provide spectroscopic fingerprint type information based on the molecular composition and structures of biologic tissues. Compared with the infrared absorption spectroscopy and fluorescence spectroscopy, Raman spectroscopy holds significant advantages such as high spatial resolution, narrow peaks, no photobleaching, and so on. In past two decades, Raman spectroscopy has been comprehensively investigated for cancer and precancer diagnosis and evaluation through various body organs include skin, breast, lung, gastric, and so forth^4^,^5^,^6^,^7^,^8^,^9^. However, a serious drawback hinders the clinical applications of Raman spectroscopy. The signal of Raman scattering is so low that only one Raman photon is produced in 10^6^–10^8^ scattering photons due to the inherently weak cross sections. The inefficient scattering requires high laser power and long collecting time for spectral acquisition, which can cause sample damage or impractical in real applications. Fortunately, with the discovery of surface-enhanced Raman spectroscopy (SERS) by Fleischman in 1974, Raman spectroscopy technique acquires huge development. When sample molecules are attached on the surface of metal nanostructure, the signal of Raman scattering can be tremendously enhanced, this phenomenon is called SERS. SERS technique has been extensively applied in the field of sensing, trace detection, and chemical analysis due to its high sensitivity^10^,^11^,^12^. It is reported that single molecule adsorbing onto a single metal nanoparticle has been successfully probed with SERS^13^.

In the field of biomedicine, SERS technique has been used for indirectly detecting tumor markers in the blood or on the cell surface by immunoassay approaches, which involves a specific interaction between an antigen and a complementary antibody^14^,^15^. Recently, label-free cancer diagnosis with blood SERS has attracted many attentions. At the early stage of cancer, the contents and conformations of biomacromolecules such as proteins, fats, nucleic acid in blood will undergo subtle changes, and these minor transformations can be revealed with SERS. Feng et al. have measured blood plasma SERS spectra of nasopharyngeal cancer patients and normal volunteers by silver nanoparticles. Combing principal component analysis (PCA) and linear discriminate analysis (LDA), they acquired the sensitivity of 90.7% and specificity of 100% for classifying nasopharyngeal cancer patients^16^. Our groups have obtained diagnostic accuracy of 98.1% for differentiation of serum SERS spectra between prostate cancer patients and normal subjects using support vector machine (SVM) technique^17^. These studies demonstrate that label-free blood SERS techniques have great potential in the field of early screening of cancer.

Owing to tiny difference of blood SERS spectra between normal subjects and patients, multivariate statistical algorithms are had to be adopted to draw effective information from several hundreds of spectral variables in measured SERS spectra. The PCA and SVM mentioned above are excellent multivariate statistical algorithms with good performance of classifying datasets. However, there are some disadvantages for PCA and SVM in processing SERS dataset. For PCA algorithms, the component spectra extracted from raw datasets by linear combination of original variables have no definite physical meaning, which make it difficult to provide effective help to understand the cancerous transformations from the molecular spectroscopy. For SVM technique, it separate classes by a hyperplane which is searched by maximizing the margin between classes^18^. With the same disadvantages, it is also difficult to pick out valuable information related to feature spectra to comprehend the cancerations from the SVM results.

To solve the problem that the diagnostic results are difficult to associate with the feature spectra by PCA or SVM, an alternative strategy is to employ feature selection technique to differentiate blood SERS spectra. Feature selection is a kind of dimensionality reduction method by mining feature subset from the original data space, while the classification attributes of the subset are as much as possible to maintain consistent with the raw data. The main advantages of feature selection are that it decreases the number of variables and helps to understand the discovered pattern by eliminating irrelevant features from raw dataset^19^,^20^,^21^. To improve the ability of digging a meaningful low-dimensional data structure in high-dimensional sample space, several feature selection strategies such as filter, embedded and wrapper have been developed according to different evaluation criteria. Genetic algorithm (GA), an adaptive heuristic search algorithm, has exhibited excellent performance of feature selection in many applications such as microarray analysis, mass spectrometry, sequence analysis, and so on^22^,^23^,^24^. GA combining with LDA technique has advantages of simplifying the classifier models, gaining new insights into variable importance, and others. This study introduces GA-LDA techniques to screening feature spectra for classification of bladder cancer patient from normal serum SERS spectra. Furthermore, the receiver operating characteristic curve (ROC) is generated to further evaluate the performance of GA-LDA. The main purpose is to explore the feasibility of using serum SERS spectra associated with GA-LDA means for noninvasive characterization and detection of bladder cancer through peripheral blood.

## Methods

### Synthesis of of AgNPs

**Figure 1:**
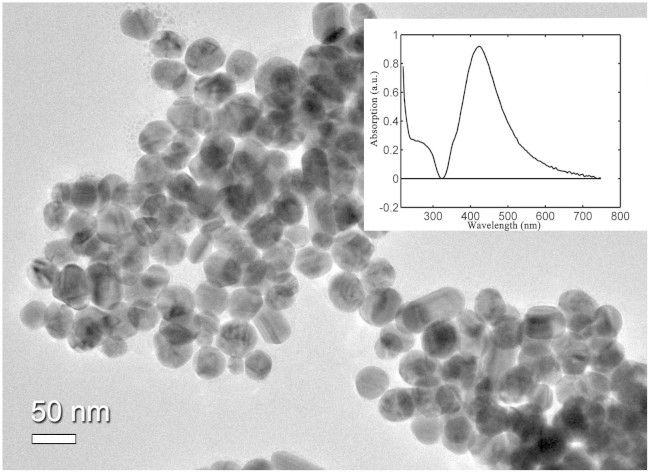
The TEM micrograph of Ag colloidal surface. The inserted picture is UV/visible absorption spectrum of the Ag colloid. The absorption maximum is located at 424 nm.

The nanosized silver particles were synthesized with the reported silver nitrate reduction method^25^. Briefly, 18 mg of AgNO_3_ was added to 100 ml water, and the solution was heated rapidly with strong stirring. When the solution was boiling, 2 ml of citrate sodium (1 wt %) was slowly added into the boiling solution. After the mixture was boiled for 40 min, the resultant silver colloids were generated and cooled in ambient conditions. A transmission electron microscopy (TEM) photograph of the prepared AgNPs is showed in figure 1. Most of the particles are spherical with good uniformity of particle size. The silver colloid particles are characterized by an absorption maximum at 424 nm in the inserted picture.

### Preparation of human serum samples

Blood serum samples were collected from 91 individuals consisting of 36 healthy volunteers and 55 bladder cancer patients who were confirmed clinically with histopathology. All patients were from Sun Yat-sen university cancer center and signed an informed consent to permit collection of blood prior to research. All experiments were approved by the medical ethics committee of affiliated hospital of Guangdong medical college. And the experimental methods are in accordance with the approved guidelines.

After 12 hours of overnight fasting, a single 3 ml peripheral blood samples were obtained from the study subjects between 7:00–8:00 A.M. After blood samples are centrifuged at 3500 rpm for 5 min, serum is obtained by removing supernatant.

Before SERS measurement, 20 μl serum was mixed with 20 μl silver colloidal nanoparticles. The mixture is stirred with the pipette tip to make it as homogeneous as possible. Then the mixture was incubated for 1 hour at room temperature. Finally a drop of this mixture was transferred onto an aluminum plate and air dried for SERS measurement.

### SERS measurements and data preprocessing

The Raman spectroscopy was recorded with a confocal Raman microscopy (Renishaw, inVia, UK) in the range of 400–1800 cm^−1^ with a spectral resolution about 1 cm^−1^. The spectra was collected in back-scattered geometry using a Leica DM2500 microscope equipped with objective 20×. A 785 nm diode laser is focused on the sample surface with a spot diameter about 5 μm and power about 0.5 mw. The software package WIRE 3.2 (Renishaw) was employed for spectral acquisition and analysis. Each Raman spectra was cumulatively acquired two times with an integration time of 10 s. All data were collected under the same conditions.

A fifth-order polynomial was employed to fit the autofluorescence background, and then this polynomial was subtracted from original spectrum. In order to compare the changes of spectral shapes and relative peak intensities among different serum samples, the area normalized under the curve was performed. The Vancouver Raman algorithms were employed to implement the baseline correction and spectral smoothing by batch processing. It is an automated autofluorescence background subtraction algorithm based on modified multi-polynomial fitting^26^.

### Genetic algorithms

Genetic algorithm is an evolutionary computing technique that can be used to solve the problems that have many possible solutions. It mimics the process of natural evolution, through a series of iterative operations, making the candidate solutions of the optimization problem approaching the optimal solution step by step^27^. GA treats an optimization by setting up a population composed of a certain number of individuals. Each individual represents a candidate solution of specific problem, and the individual is encoded with a simple chromosome-like data structure. The type of encoding varies with the difference of problems, including binary, float point, and others. When GA starts to run, it firstly generates a certain number of initial individuals, and then the individuals are evaluated by a fitness function, which is designed to assess the quality of solution for the optimal problem. The following step is to produce the offspring to form population of next generation by several types of genetic operations, which are selection, crossover and mutation. Selection operation involves picking out some outstanding individuals from the evaluated individuals according to certain rules. The selected individuals are then paired randomly with a certain probability for crossover, which is performed through intersecting chromosome fragment at the randomly selected site to yield two new individuals. After that, the mutation is carried out by altering the code of individual at the site chosen randomly with a low probability. After all of these operations finished, the new offspring is produced, and they are evaluated again for the next generic cycle, until the termination conditions are met.

In this study, the float point encoding is used. The collected serum SERS spectra contain 1270 spectral variables in the range of 400 to 1800 cm^−1^. The entire Raman spectrum is divided into 254 segments, each segment corresponding to 5 continue spectral variables. Each individual consists of 6 unique segments corresponding to 30 spectral variables. The parameters of GA are given as follow: population size of 20 individuals, mutation probability of 5%, single-point crossover probability of 70%, copy rate of 25%, and 100 generations for every run. The initial individuals were generated by randomly selecting 6 different integers less than 254. Then the individuals were evaluated through LDA methods classifying serum SERS spectra between normal subjects and cancer patients with the spectral subsets of individuals. The evaluation function is given by maximizing the accuracy of classifying serum SERS spectra. The LDA is an analysis technique that project high-dimensional samples into a lower dimensional space with the aims of maximizing the distance of inter-classes and minimizing the distance of intra-classes. The leave-one spectrum-out cross-validation is used to validate the classification performance. As such, one of Raman spectra is left out as the validation sample, the remaining Raman spectra as training set to classify the withheld Raman spectra. This process is repeated until all spectra are discriminated. The mating pool is produced by copying the outstanding individuals of 25% to instead the worst individuals of 25% based on the previous evaluation results. Then the single point crossover is performed with two different individuals selected randomly from the mating pool. During the GA iterations, the most optimal individual of each generation is completely preserved, until the appearance of new individual with bigger fitness to replace it.

## Results

**Figure 2:**
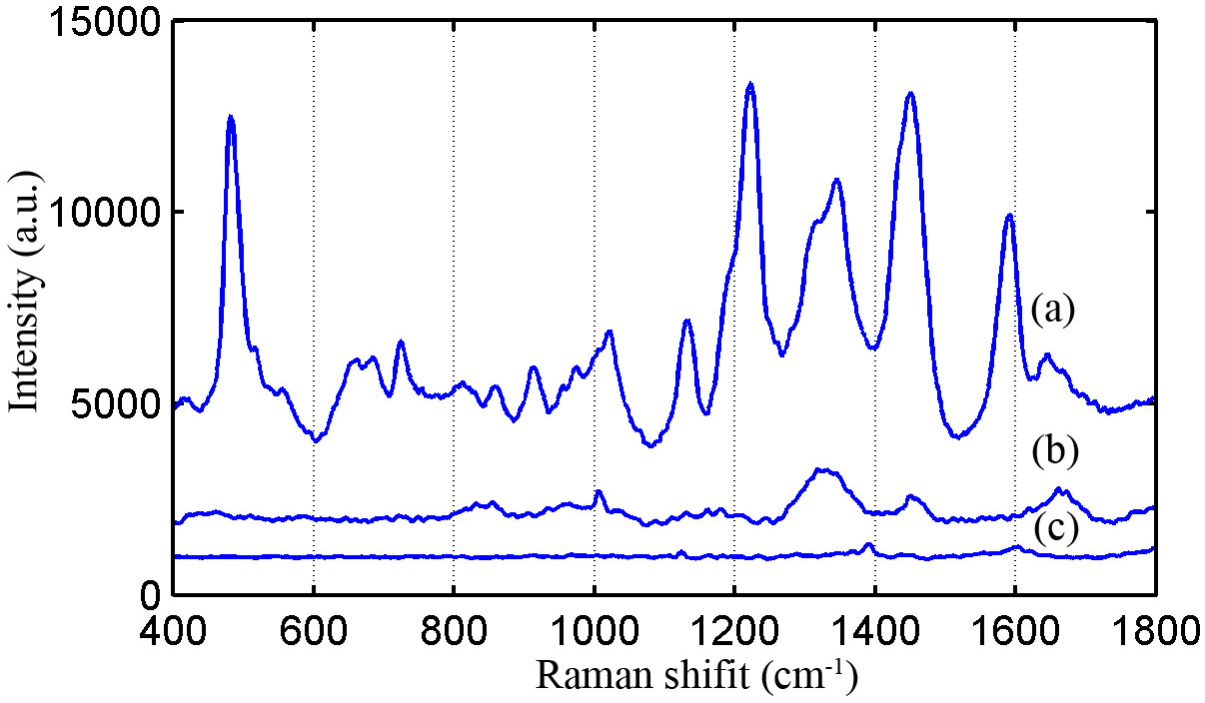
(a) Serum SERS spectra from a bladder cancer patient, (b) The regular serum Raman spectra from the same patient without Ag colloid, (c) The Raman spectra of silver colloid without serum sample.

We have measured serum SERS spectra of healthy volunteers and bladder cancer patients. In order to compare the enhanced effect of silver colloid, the regular serum Raman spectra without AgNPs and serum SERS spectra of the same patient are collected in figure 2. A huge enhancement of serum SERS spectra in many dominant vibration bands is displayed in figure 2(a), while almost no Raman peaks are observed in regular serum Raman spectra from figure 2(b). These results suggest that there are strong interactions between AgNPs and biomolecules in serum. Figure 2(c) is the Raman spectra of silver colloid without serum sample. No Raman peak is observed in the measured spectral regions, indicating no interference signal in serum SERS spectra.

**Figure 3:**
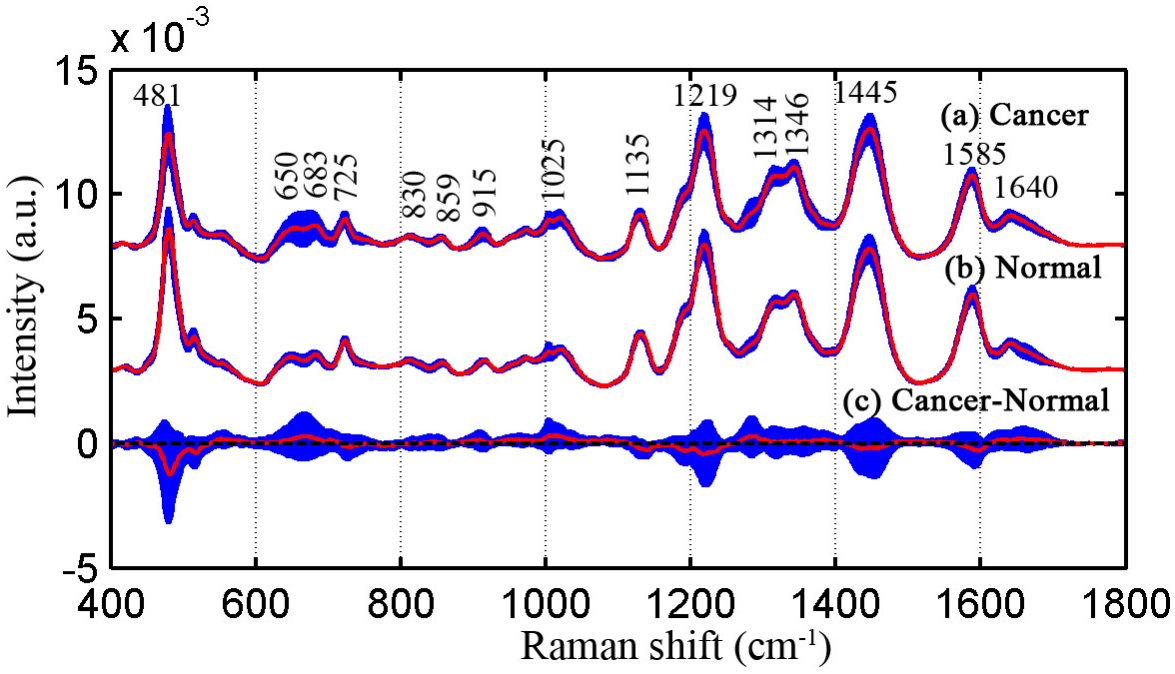
Normalized average SERS spectra of 55 cancer and 36 normal serum samples. (a) Cancer, (b) normal, (c) difference spectra of cancer-normal, shade area represents the standard deviations.

We have collected 91 serum SERS spectra from 55 bladder cancer patients and 36 healthy subjects. Figure 3 shows the normalized average SERS spectra ±1 standard deviations of bladder cancer and healthy serum in the range of 400–1800 cm^−1^. The solid line represents normalized average SERS spectra, and shade area represents one standard deviation. Figure 3(a) and figure 3(b) exhibit the serum SERS spectra of bladder cancer patients and healthy volunteers respectively. Figure 3(c) is the difference spectrum between cancer patients and healthy volunteers. The primary Raman peaks are observed in both cancer and healthy serum SERS spectra at the following peak positions with tentative biochemical assignments^6^,^8^,^16^,^28^,^29^: 481 (glycogen), 650 (C-C twisting mode, phenylalanine), 683 (C-S twist, tyrosine), 725 (C-H bending, adenine, coenzyme), 830 (Out of-plane ring breathing, tyrosine), 859 (C-C stretch of proline ring, tyrosine), 915 (C-C stretch of proline ring, glucose), 1025 (C-H stretch of phenylalanine), 1135 (C-N stretch, D-mannos), 1219 (C-C_6_H_5_, phenylalanine, tryptophan), 1314 (CH_3_CH_2_ twisting, collagen/lipids), 1346 (CH_3_CH_2_ wagging, tryptophan, adenine, guanine), 1445 (CH_2_ bending, collagen, phospholipids), 1585 (C = C bending, phenylalanine, acetoacetate, riboflavin) and 1640 cm^−1^ (C = O stretch, amide I, α-helix proteins). The strongest peaks are at 481, 1135, 1219, 1445 and 1585 cm^−1^. The spectral difference in figure 3(c) displays that the normalized intensity of SERS peaks at 683, 1025, 1314 and 1640 cm^−1^ are more intense for cancer serum than for healthy serum, while Raman bands of 481, 1219, 1445 and 1585 cm^−1^ are higher in normal serum samples. The changes of Raman spectral intensity indicate that there is an increase or decrease in the percentage of a certain type of biomolecules relative to the total SERS-active constituents in serum associated with neoplastic transformation. The differences in serum SERS spectra between bladder cancer patients and normal subjects suggest there are potential to screening bladder cancer with serum SERS spectra.

**Figure 4:**
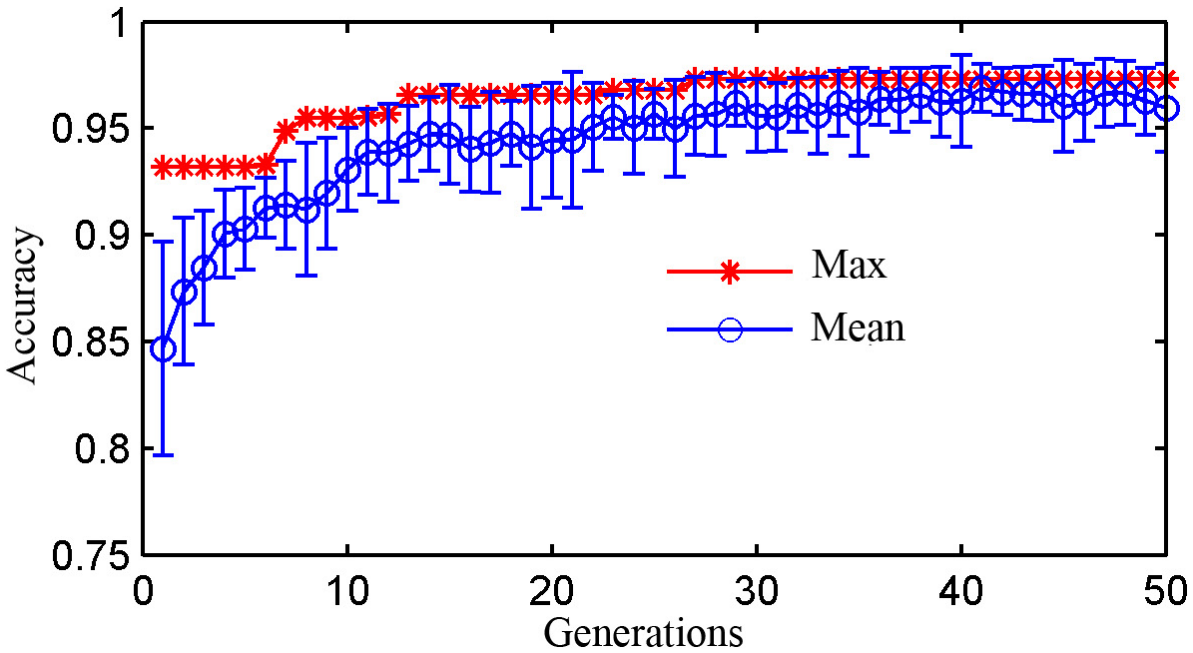
The mean classification accuracy of the population ± 1SD versus the best performance individuals in 50 generations with GA-LDA. (20 individual, cross rate = 0.7, mutation rate = 0.05).

It is clearly displayed from figure 3(a) and (b) that the spectral profile of cancer patients and healthy subjects is very similar, suggesting the necessities of using a powerful data analysis algorithm to acquire the effective information for differentiating the normal and cancer serum SERS spectra. In this study, the GA-LDA algorithms are employed to search for the significant Raman feature spectra that are relevant to bladder canceration. The parameters of GA are adjusted repeatedly to acquire the significant spectral feature for classification. Figure 4 shows the best and mean ± 1SD classification accuracy of population in 50 generations at one run with GA-LDA algorithms. It is obvious that the maximal accuracy no more increases after 26 generations, while the mean accuracy of population is greatly improved.

**Figure 5:**
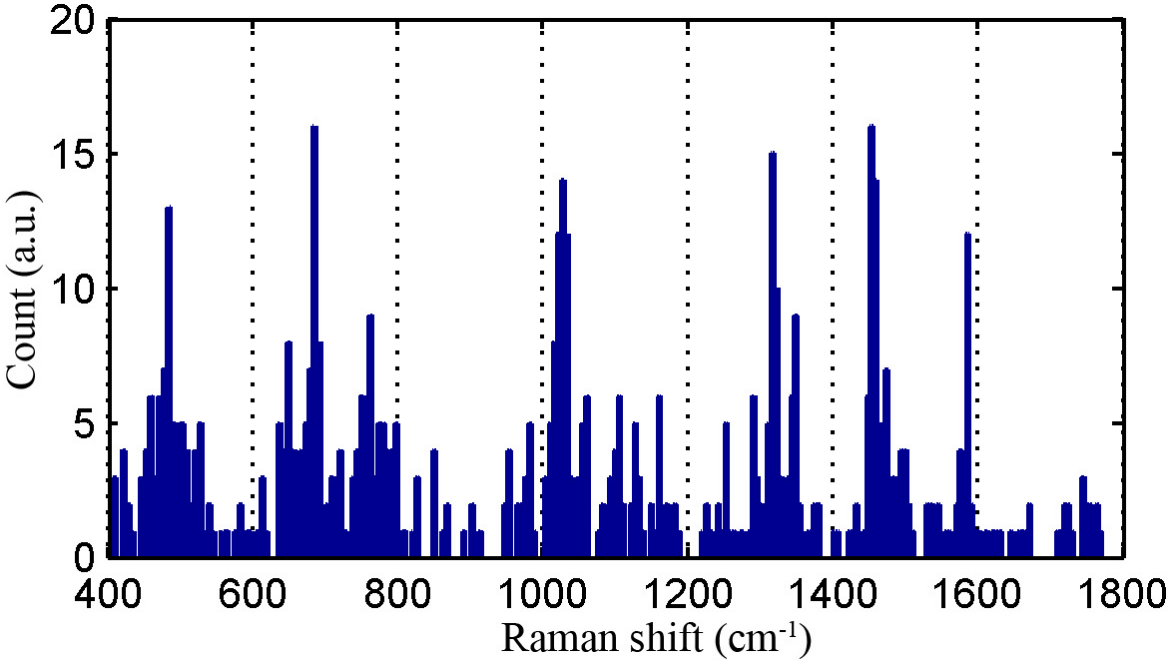
The cumulative counts of Raman bands chosen with GA-LDA in 100 runs.

In order to dig significant feature information from serum SERS spectra, the GA-LDA algorithms are independently run 100 times and the searched Raman variables of each run are cumulatively counted. The Raman bands with maximum counts are supposed to be significant diagnostic feature for identifying bladder cancer from healthy serum SERS spectra. Figure 5 displays the cumulative count frequencies of Raman variables searched by GA-LDA algorithms in 100 runs. The significant Raman shifts with the highest cumulative counts lie in the range of 481–486, 682–687, 1018–1034, 1313–1323, 1450–1459 and 1582–1587 cm^−1^. The tentative vibrational assignments of biochemical compositions in serum are listed in Table 1^16^,^30^. The intensity differences between the normal and cancer serum among these searched Raman spectral features are verified to be significant for classification of bladder cancer.

**Figure 6:**
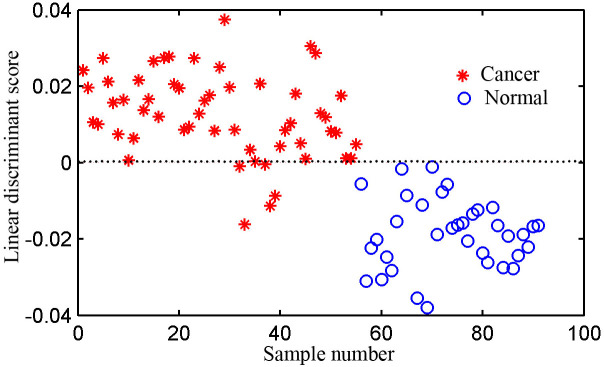
Scatter plot of the linear discriminant scores of bladder cancer and normal serum SERS spectra using GA-LDA.

The LDA discriminated model is then built with the six significant Raman bands together with the leave-one spectrum-out cross-validation. Figure 6 is the scatter plot of the linear discriminant scores of normal and cancer serum SERS spectra using GA-LDA diagnostic model based on the six significant Raman bands together with the leave-one spectrum-out cross-validation. With the separation line in scatter plot, the GA-LDA diagnostic model produces diagnostic sensitivity of 90.9%, specificity of 100% and overall accuracy of 94.5% for discriminating bladder cancer from normal serum. The results suggest that the GA-LDA algorithms develop a novel way to characterize and classify bladder cancer patients from serum SERS spectra of normal volunteers.

**Figure 7:**
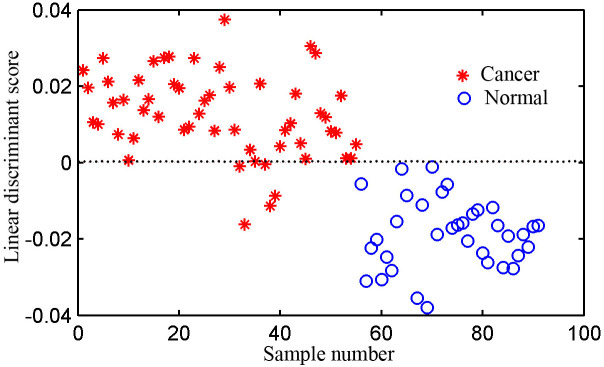
Receiver operating characteristic (ROC) curves of serum SERS spectra for classifying bladder cancer patients and normal subjects with PCA-LDA and GA-LDA algorithms together with the leave-one spectrum-out cross-validation technique. The integration area under the ROC curves for PCA-LDA and GA-LDA are 0.920 and 0.986, respectively, illustrating the efficient performance of GA-LDA in bladder cancer diagnosis with serum SERS.

To further evaluate the performance of LDA discriminant model built with the six significant Raman bands, the ROC curve is generated in figure 7 from the LDA scatter plot of figure 6. ROC curve is a plot of the true positive rate against the false positive rate with the different diagnostic thresholds. The integration area under the ROC curves (AUC) positively correlates with the diagnostic accuracy. The larger AUC represents the greater forecast accuracy for the test classifier. The AUC value is 0.986 for the classifier built with GA-LDA algorithms. To comparatively assess the diagnostic performance of GA-LDA-based algorithm, the PCA algorithms are applied to the same serum SERS spectra dataset. The diagnostic sensitivity of 74.6%, specificity of 97.2% and overall accuracy of 83.5% are acquired with the first 20 principal components, which account for cumulative contribution rate 93.9% of the total variance together with the leave-one spectrum-out cross-validation. The ROC curve of PCA-LDA-based algorithms is generated in figure 7 with AUC value of 0.92. These results demonstrate that the GA-LDA-based diagnostic algorithms are superior to the PCA-LDA-based algorithms in the diagnostic performances.

## Discussion

Currently cancer has become one of most deadly diseases to threat human lives. The early screening cancer with a non-invasive method is an important field of medical studies. Bladder cancer has an unusually high propensity of recurrence after treatment. The periodic health examination with nondestructive means is essential for these who have removed the bladder tumor^31^. As a unique non-invasive detection technique Raman spectroscopy has drawn extensive attentions. It has been applied to detect various human organs and acquired many interesting results. Several groups have carried out the researches of diagnosing bladder cancer with Raman spectroscopy. Stone et al. have reported that Raman microscopy can provide sensitivities and specificities >90% in an eight-group algorithm for bladder tissue samples^32^. Soon after they measured the Raman spectroscopy of snap-frozen bladder samples by a fiber-optic based clinical Raman system. With the constructed diagnostic algorithms, they acquired an overall accuracy of 84% for differentiating benign and malignant bladder samples^33^. Recently Draga et al. employed a high-volume based Raman probe to investigate the potential of determining the invasiveness of bladder caner. The diagnostic sensitivity of 85% and specificity of 79% are obtained^1^. Barman group used a confocal Raman probe to diagnose bladder cancer, and they acquired an improved sensitivity of 85.7%, specificity of 100%^34^. However, the weak Raman signal and short depth of penetration of light in tissues hinder clinical applications of Raman spectroscopy. With the discovery of SERS, Raman spectroscopy acquires rapid developments. Because SERS can provide ultrasensitive characterization down to single molecular level, it has aroused more expectations in the field of cancer diagnosis.

Blood is an idea material for non-invasive cancer screening with a way of implementing conveniently and repeatedly, and the sampling and testing even can be executed continuously in the course of monitoring high risk patients. At the early stage of cancer, the contents and conformations of blood components such as proteins, lipids will generate subtle changes which can be disclosed with SERS. In this study, we have measured the serum SERS spectra of bladder cancer patients and healthy volunteers. The collected regular serum Raman spectra without silver nanoparticles and serum SERS spectra of the same patient are displayed in figure 2. The dramatic increase of SERS spectra intensity in many dominant bands suggests there is a great potential to diagnose the bladder cancer by serum SERS technique. The specific differences in figure 3(c) reflect the changes of some biomolecules relative to the total SERS-active constituents in serum associated with malignant transformation. For example, the Raman peak 481 cm^−1^ (glycogen) is found to decrease significantly, indicating a reduction of glycogen content relative to the total Raman-active composition in the serum of bladder cancer patients. The likely cause involves the abnormal glucose metabolism in cancer tissue^35^. The Raman peak of 1640 cm^−1^ (C = O stretch, amide I, α-helix proteins) shows a higher percentage of signals for the bladder cancer serum as compared to the normal serum. It is likely related to elevated percentage of histones concentration with respect to the total SERS-active constituents in cancer serum^28^,^36^. Berpholt et al. also find that the Raman signal of esophageal cancer tissue mainly associated with the increase of DNA and histones, and the decrease of the glycogen, collagen, triolein and actin^36^. But an important Raman peak of 1004 cm^−1^ attributed to phenylalanine in tissue doesn't appear in serum SERS spectra, indicating without enhancement of 1004 cm^−1^. However, the measured serum SERS spectral profile is similar for bladder cancer patients and normal subjects. It is necessary to develop the sophisticated multivariate diagnostic algorithms to interpret these minor spectral changes.

A conventional algorithm is to use PCA, which belongs to feature extraction technique. Although PCA can provide satisfactory results for classifying high dimensional dataset, it has a serious disadvantage that the principal components extracted from raw dataset have no defined physical meaning. In this work, the artificial intelligence technique, genetic algorithm combined with linear discriminant analysis, is introduced to search the significant Raman bands for classifying serum SERS spectra. Genetic algorithm is a kind of feature selection technique that digs subset of feature variables to construct classifier models. The main advantages of GA involve that it improves model interpretability through providing simplified diagnostic models, and that it enhances the performance of learning models by removing most irrelevant and redundant features from the original data. The important six spectral bands in the regions around 481–486, 682–687, 1018–1034, 1313–1323, 1450–1459 and 1582–1587 cm^−1^ are picked out with the GA-LDA. These Raman bands mainly correlate with dysplasia progression. For example, the Raman bands 682–687 cm^−1^ and 1313–1323 cm^−1^ assigned nucleotide chains are higher in cancer serum than in normal serum, indicating the percentage of nucleic acid contents relative to total serum SERS-active components is increased in bladder cancer serum. The results are consistent with the diagnostic feature of cancer in histopathology, an increase of the nuclear-cytoplasmic ratio due to DNA aneuploidy. Stone N et al. report that the Raman microscopy spectra of bladder cancer tissue are characterized by a decrease of collagen I and III and an increase of lipids and DNA^37^. Draga et al also find that the Raman spectra of bladder cancer are significantly expressed by elevated intensities of nucleotide chains, and that malignant bladder tissue contains lower concentrations of collagen and increased concentrations of other specific proteins^2^. The reason for the increase of nucleic acids content in serum remains unclear. It is speculated that maybe the apoptosis and necrosis release their subsequent lysis in the blood^16^. The Raman bands 1450–1459 cm^−1^ (CH2 bending, collagen/lipids) show a considerable decrease in cancer serum, revealing the reduction of the proportion of fat content. The likely reason may be that the tumor's vigorous metabolism consumes a lot of fat, resulting in decrease of lipid molecules^17^.

Through the classifier models built with identified Raman bands, the diagnostic sensitivity of 90.9% and specificity of 100% are acquired for the differentiation of serum SERS spectra between bladder cancer patients and normal subjects. To further assess the performance of diagnostic models built with GA-LDA, the ROC in figure 7 is generated from the scatter plot of the linear discriminant scores in figure 6. The integration area of 0.986 under the curve indicates the efficient performance of diagnostic models. To compare the performance of GA-LDA, the regular multivariate statistic algorithms of PCA-LDA are employed to process the same serum SERS spectra dataset. The diagnostic sensitivity of 74.6%, specificity of 97.2% and accuracy of 83.5% are obtained by the first 20 principal components accounting for cumulative contribution rate 93.9% of the total variance together with the leave-one spectrum-out cross-validation. The results are lower than these obtained with GA-LDA. The AUC value of 0.920 in figure 7 further comparatively confirms the efficient performance of diagnostic models developed with GA-LDA. Obviously, compared to the PCA-LDA technique, the GA-LDA algorithms provide a simplified diagnostic model and improved diagnostic accuracy by identifying the specific Raman bands relation to the biochemical diagnostic information. These results demonstrate that serum SERS spectra associated with GA-LDA algorithms have great potential to non-invasively characterize and identify bladder cancer.

The improved diagnostic performance of GA-LDA model may be attributed to two reasons. One hand, the GA is a heuristic optimization algorithm that possesses inherent ability of exploiting the mutual interactions among spectral variables according to their variables importance. Therefore the GA-LDA feature selection technique can acquire the diagnostically significant biochemical changes associated with carcinogenesis transformation in serum^30^. On the other hand, PCA belongs to feature extraction technique that extracts information from full spectrum, while many redundant data and noise within the entire Raman spectrum will also contribute to the principal components. These poisonous contributions depress the performance of the PCA. There are several reports about the applications of feature selection technique in Raman spectroscopy. Huang groups used ACO and LDA to identify the biochemical important Raman bands from normal and neoplastic stomach tissue Raman spectra. They acquired diagnostic sensitivity of 94.6% and specificity of 94.6% for distinction of gastric neoplasia from seven diagnostically important Raman bands^28^. Our groups previously have applied GA-LDA algorithms to search significant Raman bands for noninvasive detection of nasopharyngeal tumor from the normal and neoplastic nasopharyngeal tissues Raman spectra. A diagnostic sensitivity of 69.2% and specificity of 100% for distinction of nasopharyngeal neoplasia is provided by the diagnostic models developed with identified seven Raman bands^38^.

In this study, we have successfully characterized and classified the serum SERS spectra of bladder cancer patients and normal subjects by GA-LDA technique. The interesting results suggest a potential application of noninvasive bladder cancer screening by peripheral blood samples. But there are several disadvantages for our researches. First, the patients who provide blood samples are almost in middle or advanced cancer for our study, so the diagnostic models built with these serum samples belong to corresponding cancer staging. Whether these models can be applied to diagnose early cancer remains unknown. The second, owing to the limit of sample numbers and diversities, maybe there are some differences between the identified six Raman bands and real feature spectra. To resolve these problems, we will further carry out in-depth research in the future work.

## Conclusions

In conclusion, we have measured serum SERS spectra of normal subjects and bladder cancer patients. with the artificial intelligence technique GA-LDA, six important Raman bands associated with proteins, nucleic acids and lipids in the region of 481-486, 682–687, 1018–1034, 1313–1323, 1450–1459 and 1582–1587 cm^−1^ are picked out from the measured serum SERS spectra. An improved diagnostic accuracy of 94.5%, sensitivity of 90.9% and specificity of 100% is obtained for classifying serum SERS spectra between bladder cancer patients and normal subjects by the diagnostic models built with identified six Raman bands. Compared with the PCA-LDA algorithm, the GA-LDA algorithm is a very attractive technique that is expected to provide a more accurate diagnostic model for the clinical application of Raman spectroscopy. This study demonstrates that the serum SERS associated with GA-LDA technique has enormous potential to characterize and non-invasively detect bladder cancer through peripheral blood.

## Author Contributions

S.L. and J.X. designed the experiments. L.L. and Q.Z. collected and processed human blood samples. Z.L., M.J., C.S. and L.L. performed experiments. Z.G., S.L. and J.X. analyzed the experimental data. S.L. and L.L. designed the programs. S.L. and Y.Z. wrote the main manuscript text. All authors discussed the results and commented on the manuscript.

## Figures and Tables

**Table 1:** Tentative assignments of significant Raman bands identified by GA-LDA algorithms^16^,^30^

Peak position (cm^−1^)	Vibrational assignments	Intensity change (cancer-normal)	*p*-value
481–486	glycogen	−	3.13E-6
682–687	C-S twist, tyrosine, DNA (guanine)	+	2.24E-4
1018–1034	C-H stretch of phenylalanine	+	5.71E-5
1313–1323	CH_3_CH_2_ wagging of proteins/nucleic acids	+	1.14E-3
1450–1459	CH2 bending, collagen/lipids	−	1.36E-3
1582–1587	C = C bending, phenylalanine, acetoacetate, riboflavin	−	1.15E-3

Note: The mean intensity changes (increase:+; decrease:−) and the *p*-values of unpaired two-sided Student's t-test on the SERS Raman band intensities of normal and bladder cancer serum.
